# Dietary Heme Iron: A Review of Efficacy, Safety and Tolerability

**DOI:** 10.3390/nu17132132

**Published:** 2025-06-27

**Authors:** Douglas Kalman, Susan Hewlings, Alexis Madelyn-Adjei, Blake Ebersole

**Affiliations:** 1 Substantiation Sciences, LLC, Weston, FL 33332, USA; dkalman@substantiationsciences.com (D.K.); sue.hewlings@substantiationsciences.com (S.H.); 2 Nutrition Department, Dr. Kiran C. Patel College of Osteopathic Medicine, Nova Southeastern University, Fort Lauderdale, FL 33328, USA; 3 School of Medicine, Anglia Ruskin University, Chelmsford CM1 1SQ, UK; 4 NaturPro Scientific, LLC, Carmel, IN 46032, USA; blake@npscientific.com

**Keywords:** iron, heme, nutrition, hemoglobin, ferritin

## Abstract

Iron is a fundamental micronutrient essential for oxygen transport, enzymatic activity, and metabolic homeostasis. Yet it remains the most deficient nutrient in the world, with more than 2 billion people estimated with iron deficiency anemia. In the diet, animal foods provide iron primarily as heme iron. Dietary heme iron is absorbed through the active transport pathways catalyzed by heme oxygenase in the intestinal enterocyte. This form of heme differs in its bioavailability, absorption mechanisms, and tolerability compared to non-heme forms of iron, including iron salts and chelates. Adding more heme iron to a diet, including through iron supplements, may help to reduce the prevalence of iron deficiency. Future research should focus on research of heme iron supplementation strategies to enhance absorption efficiency, gut microbiome health, and safety, ensuring optimal iron status across diverse populations.

## 1. Introduction

Iron is an essential nutrient and a critical component of hemoglobin, necessary for various metabolic processes including oxygen transport, immunity, cell function, and gene expression [1,2,3]. Dietary iron requirements increase in response to factors such as body weight, muscle mass, blood loss, hypoxia, pregnancy, and periods of rapid growth [4].

Iron exists in two primary forms in the diet: heme iron, derived from animal sources such as meat and fish, and non-heme iron, predominantly found in plant-based foods and widely used in iron supplements [3]. These two forms exhibit distinct absorption mechanisms, with heme iron being more bioavailable and less influenced by dietary inhibitors than non-heme iron [2,3].

Iron deficiency (ID) and iron deficiency anemia (IDA) are widespread global health concerns and common conditions in clinical practice. ID is one of the most prevalent nutritional deficiencies globally, affecting approximately 27% of the world’s population [1,3,5,6,7]. Iron deficiency is typically identified through low serum ferritin (<30 ng/mL in men, <15 ng/mL in women) and transferrin saturation (Tsat < 20%), reflecting depleted iron stores without necessarily lowering hemoglobin levels [1,2]. Iron deficiency is associated with symptoms such as fatigue and shortness of breath, and if left untreated, may increase the risk of morbidity and mortality.

In contrast, IDA develops when prolonged iron depletion results in hemoglobin levels dropping below the diagnostic threshold, leading to impaired oxygen transport and anemia-related symptoms [3]. The World Health Organization (WHO) defines IDA as a hemoglobin (Hb) level below 13 g/dL in men and 12 g/dL in women, marking the transition from iron depletion to anemia. The highest rates of IDA are in women and children, where it contributes to the risk of low birth weight, pre-term delivery, and cognitive impairment in infants and children [3,8].

In the United States, IDA is a recognized public health concern, as highlighted by NHANES data from 2003 to 2012, which identified vulnerable subgroups including African Americans, Latin Americans, individuals over the age of 60, breastfed infants, vegetarians, pregnant women, and women of reproductive age [8,9]. An increase in mortality has mirrored the rise in iron deficiency in the U.S. Of food items in the USDA database with revised concentrations from 1999 to 2015, 62% reported lower concentrations of iron. During this time, dietary iron intake decreased by 7% and 10% for males and females in the U.S., respectively. Likewise, iron status markers were shown to decrease during this time [10].

Furthermore, recent findings indicate that approximately 30% of American children aged 1–2 years are classified as iron deficient [11]. If left untreated, IDA can progress to microcytic anemia, leading to quality-of-life symptoms such as fatigue, weakness, difficulty concentrating, depression, decreased productivity, and restless leg syndrome, as well as adverse pregnancy outcomes like low birth weight or pre-term delivery.

Iron insufficiency, which often precedes clinical deficiency, affects nearly half of the general American population, disrupting metabolic processes, increasing chronic disease risk, and negatively impacting overall quality of life and physical and mental performance [3,8,9]. Given its critical role in human metabolism and the widespread prevalence of deficiency, understanding dietary sources and supplementation options for iron is vital to addressing this public health challenge.

This review examines the role of heme iron supplements, emphasizing their differentiation from non-heme iron and the potential to address iron deficiency and insufficiency with improved efficacy and tolerability.

## 2. Dietary Sources of Iron

**Figure 1:**
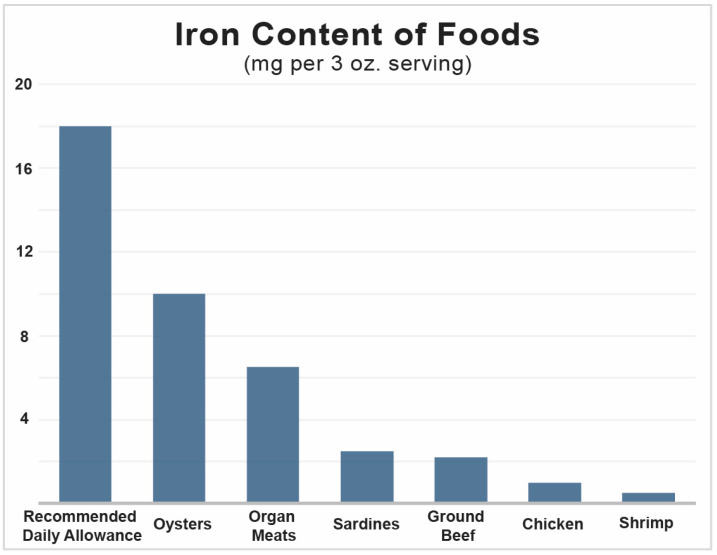
Recommended Daily Allowance (RDA) for iron (18 mg) and the iron content of common foods. One serving of common animal proteins such as ground beef and chicken contains a fraction of the RDA, one indication that it can be challenging to meet iron intake (USDA).

The iron content of food varies depending on the source, with beef and fish providing 1–3 mg per 100 g (Figure 1, [3]). In developed countries, red meat consumption primarily meets iron needs, whereas plant-based consumption predominates in non-developed countries [1,4]. In some countries, supplementation contributes to iron intake. Among Americans, 14–18% take iron supplements, with the highest usage observed in pregnant (72%) and lactating women (60%) [5].

### 2.1. Two Main Forms of Dietary Iron

**Figure 2:**
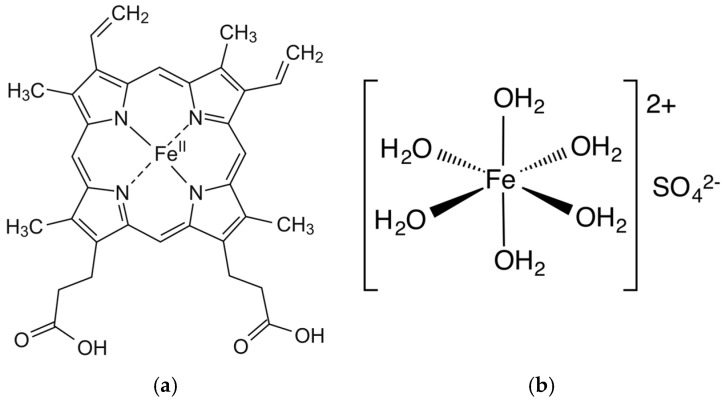
Molecular structures of heme iron (**a**) and iron sulfate (**b**).

Iron exists in two main dietary forms: heme iron and non-heme iron. Heme iron, derived from animal-based sources such as meat, poultry, and seafood, is highly bioavailable, with absorption rates of 25–30%. In contrast, non-heme iron, predominantly found in plant-based foods such as grains, legumes, and vegetables, has a much lower absorption rate of approximately 3–5%. Heme iron’s superior absorption efficiency is attributed to its distinct uptake pathway that appears to be mostly unaffected by dietary inhibitors (Figure 2). Conversely, non-heme iron absorption is strongly influenced by inhibitors like phytates and other minerals, and enhancers like ascorbic acid and gastric acid [6,7].

Two sources of dietary iron exist in food: heme and non-heme [1]. Heme iron is primarily present in hemoglobin and myoglobin in seafood, poultry and meat [1,6,8]. Heme iron is 2–3 times more bioavailable than non-heme, and accounts for 10–15% of the iron consumed from food.

### 2.2. Dietary Iron for Fortified Food and Supplements

Non-heme iron is consumed in various forms, including iron sulfate, iron fumarate, and iron chelates, which are subject to monograph review by the United States Pharmacopeia (USP) and other regulatory standards such as the Food Chemicals Codex (FCC). These compounds are derived from refined earth minerals and, in the case of chelates, are bound to isolated amino acids to enhance bioavailability [9,10].

Plants, such as fruits, vegetables, grains, and beans, are sources of non-heme iron. Plant-based iron ranges from 0.2 to 3.6 mg per serving, with spinach providing approximately 3.6 mg per 100 g, and beans offering around 1.4 to 2.1 mg per serving, depending on the variety [1,5,6].

Non-heme iron, present in plant-based foods like fruits, vegetables, nuts, seeds, grains, and beans, comprises 85–90% of dietary iron intake but has lower bioavailability compared to heme iron [1,4,7,8].

### 2.3. Challenges of Iron Supplementation for Iron Deficiency

Data shows that iron supplementation is more effective at treating IDA than dietary (food-only) interventions [11]. However, readily accessible oral iron supplements often cause side effects like dyspepsia and constipation, with severity increasing at higher doses [12]. High-dose iron supplementation can also lead to oxidative stress [13,14].

Excess iron supplementation poses risks to infants with normal hemoglobin levels, and while iron therapy can improve growth in deficient children, it may reduce growth and weight gain in iron-replete children [15,16]. In addition, excessive iron may compete with the absorption of other nutrients. Therefore, there is a need to explore options for overcoming the side effects. Likewise, several challenges exist for iron supplements to correct widespread iron deficiency, meet public nutritional needs without the potential for harm, and prevent iron overload or toxicity in individuals during chronic periods of supplementation. Addressing the limitations of traditional iron supplementation requires exploring alternative strategies, such as leveraging the unique advantages of heme iron and its ability to enhance non-heme iron absorption—a phenomenon referred to as the ‘meat factor.’

## 3. Heme Iron and the “Meat Factor”

Heme iron not only provides superior bioavailability on its own but also enhances the absorption of non-heme iron through a phenomenon known as the “meat factor.” This effect, observed in studies such as Layrisse et al.’s [17] evaluation of maize and black bean meals, demonstrates that even small amounts of heme iron can significantly improve the absorption rates of non-heme iron (Table 1) [18]. Combining heme iron with non-heme iron supplementation increased total absorption by 40% [19].

### 3.1. Advantages of Heme Iron Supplementation

Heme iron supplementation offers distinct advantages over non-heme iron. Unlike non-heme iron salts, which cause unpleasant gastrointestinal disturbances at high doses, heme iron achieves optimal absorption at lower doses and is not associated with unabsorbed iron toxicity.

The greater bioavailability of iron from animal foods as heme iron contributes to a larger effect on serum ferritin, among other key iron markers. Serum ferritin, a key indicator of iron stores, is consistently higher in individuals consuming meat compared to vegetarians, largely due to the superior bioavailability of heme iron. For example, a systematic review and meta-analysis encompassing 24 cross-sectional studies found that adult vegetarians had significantly lower serum ferritin levels than non-vegetarians, with a mean difference of −29.71 µg/L (*p* < 0.01) [21].

In a Dutch cohort study of 2323 blood donors, higher heme and lower non-heme iron intake were associated with elevated hemoglobin and serum ferritin levels, illustrating the critical role of dietary iron composition in maintaining iron stores [22]. Similarly, a cross-sectional study in Australia involving 299 healthy premenopausal women demonstrated positive associations between heme iron intake and ferritin levels [23]. These findings support the key contribution of heme iron to serum ferritin levels and its physiological advantages over non-heme iron.

A study conducted in Sweden among teenage girls revealed that omnivores had significantly higher ferritin levels (average 19.6 µg/L) compared to vegetarians and vegans (average 10.9 µg/L [24]. Furthermore, Australian research highlights that while vegetarians can meet iron needs through plant-based sources, the lower bioavailability of non-heme iron often results in reduced ferritin levels compared to meat-eaters [25].

The SU.VI.MAX study found meat and fish intake among French women contributed to higher iron status [26]. In this study, 22% of the women had depleted iron stores and 4% experienced iron-deficiency anemia—yet dietary iron intake was below recommended levels for 93% of women, with heme iron accounting for only 10% of their intake. Corroborating other evidence on heme iron intake and ferritin levels, serum ferritin correlated positively with meat and fish consumption, but negatively with dairy, calcium, and fiber—emphasizing the role of dietary quality in iron status [26].

### 3.2. Iron Absorption and Metabolism

The majority of plant-derived iron is non-heme and when consumed is bound within the plant cell–matrix to be extracted by the digestive system. To better understand the factors influencing iron uptake, it is essential to look into the distinct mechanisms underlying heme and non-heme iron absorption. Heme and non-heme iron are absorbed through distinct mechanisms, with heme iron uptake remaining unaffected by the presence of non-heme iron due to separate intestinal transport pathways [27].

While heme iron absorption is efficient and resistant to dietary inhibitors such as phytates, non-heme iron absorption is influenced by both enhancers and inhibitors present in the diet. Non-heme iron, which comprises the majority of plant-derived and supplemental iron, is often bound within cellular matrices (as plants)—or as salts and chelates in supplements, such as ferrous sulfate and fumarate. Non-heme salt and chelated forms, introduced in the 20th century, have shown effective in improving iron status when administered in doses of 30–65 mg elemental iron daily. Yet, these forms are not without challenges, due to lower absorption rates and gastrointestinal side effects [7].

### 3.3. The “Iron Fork” in the Road

**Figure 3:**
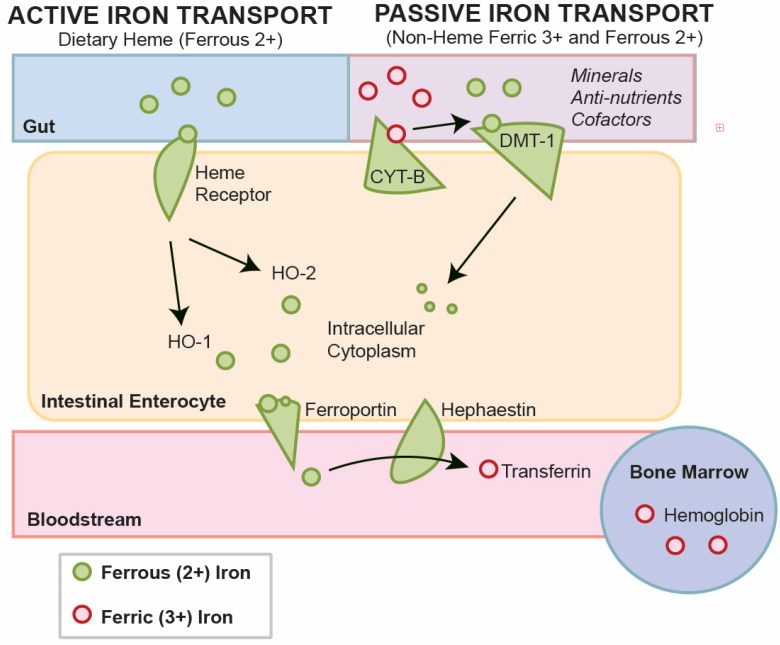
Iron absorption and transport mechanisms. Heme iron, sourced from animal foods, is actively absorbed intact into intestinal enterocytes. Heme iron bypasses dietary inhibitors to the enterocyte, where heme oxygenase re-leases ferrous iron (Fe^2+^). This is then exported into the bloodstream through ferroportin. Alternatively, non-heme iron, predominantly from synthetic or plant-based foods, often initially exists as ferric iron (Fe^3+^), requiring reduction to ferrous iron (Fe^2+^) by duodenal cytochrome B (DcytB) before approaching the competitive divalent metal-ion transporter 1 (DMT1). Absorption of non-heme iron is influenced by dietary enhancers like ascorbic acid and inhibitors such as polyphenols, phytates, and calcium, resulting in lower bioavailability compared to heme iron.

Iron absorption primarily takes place in the duodenum and jejunum of the small intestine, facilitated by two distinct pathways for heme and non-heme iron (Figure 3). These pathways ensure adequate iron uptake for metabolic functions—but differ significantly in their absorption efficiency, mechanisms, and regulatory frameworks [6,8].

### 3.4. Multiple Facets of Heme Iron Absorption

Heme iron is absorbed intact, through specialized transporters. Its absorption is minimally influenced by dietary inhibitors, such as phytates, tannins, and oxalates, making it highly bioavailable. After absorption into enterocytes, heme iron is degraded by heme oxygenase to release ferrous iron (Fe^2+^), which is then exported into circulation through ferroportin (FPN1) [6,28]. Heme iron, in its native form, is bound in the heme molecule and is soluble at neutral pH. Because iron is bound to the heme porphyrin molecule, its iron is more easily absorbed into the enterocyte [1,12]. Heme iron is released from hemoproteins, with the help of gastric acid and proteolytic enzymes [8].

Heme iron is 200–400% more bioavailable than non-heme forms of iron. In iron-sufficient and iron-deficient women, heme iron was significantly better absorbed (16% vs. 4.6% and 22% vs. 9.5%, respectively) [29]. The absorption of heme iron is efficient and largely uninfluenced by other dietary constituents [12]. Heme iron absorption occurs through a separate pathway than non-heme iron, and therefore it does not compete with non-heme iron absorption [30]. Iron availability in the colon lumen is a critical signal for the expression of virulent genes by pathogens and hosts. It has been shown that a ferroportin-mediated efflux of iron, and consequent changes in the amounts of available iron to *Salmonella* [31] fosters a pathogenic response.

Improved iron utilization via dietary heme iron in combination with non-heme iron sources has been evaluated. In one comparative study, concluded that in both pregnant and non-pregnant women, iron utilization was more efficient, as leading to less sensitivity to hepcidin and iron stores compared with ferrous sulfate [32]. Likewise, the presence of heme iron in the matrix of animal food, including fish, pork, chicken, lamb and beef has been shown to enhance non-heme iron absorption [17,29]. This phenomenon is known as “meat factor” [1,19].

### 3.5. The “Meat Factor” of Heme Iron

The synergistic role of heme iron in enhancing non-heme iron absorption is popularly referred to as the “meat factor.” This phenomenon occurs when peptides or proteins from animal tissues maintain non-heme iron in a soluble form, improving its uptake efficiency. Studies nearly 60 years ago started the pattern of studies on iron status from animal diets providing heme iron. The first in 1968 found adding heme iron to meals containing non-heme iron can increase overall iron absorption by up to 150% [17,18,19].

Layrisee et al. [17] found a 150% increase in iron absorption in humans when heme iron-containing fish and veal were added to plant-based meals of maize and black beans. Later, Hurrell et al. conducted a study to evaluate the impact of the meat factor on iron absorption; the findings were similar to those of other researchers supporting increases in iron absorption with the presence of fish or other types of meat [18]. The mechanism of the “meat factor” is fairly well understood. Iron, in its elemental form, is reactive. Evolution has provided clues on how humans can get enough, which appears to be partially by eating other animals. The dedicated transport pathway for heme iron through heme oxygenase is part of a highly regulated system developed to limit iron toxicity. The influence of bound heme iron, in addition to the encapsulating porphyrin matrix which contains iron, serves as a kind of nature-provided benchmark for the sustained, everyday fortification and supplementation of iron [12,33].

## 4. Bioavailability Challenges in Iron Supplements

Iron supplementation plays a crucial role in managing iron deficiency; however, differences in bioavailability, tolerability, and absorption mechanisms across supplement forms present notable challenges. Some iron supplements exhibit low bioavailability and gastrointestinal side effects due to unabsorbed iron accumulating in the gut, leading to oxidative stress, inflammation, and reduced compliance [12,33].

Non-heme iron, primarily found in plant-based foods, exists in the ferric (Fe^3+^) state and requires reduction to ferrous iron (Fe^2+^) via duodenal cytochrome B (DcytB). This reduced form is transported into enterocytes through divalent metal-ion transporter 1 (DMT1). Non-heme iron absorption is significantly affected by dietary enhancers such as ascorbic acid and inhibitors like polyphenols, phytates, and calcium, resulting in lower bioavailability [4,13]. Phytates and polyphenols form insoluble complexes that reduce absorption, while ascorbic acid and gastric acid improve non-heme iron uptake by promoting its reduction to the more absorbable ferrous form [2]. Heme iron, in contrast, is absorbed through a separate mechanism and remains unaffected by dietary inhibitors, making it a more efficient form of iron intake.

Calcium intake has been questioned as a potential inhibitor of iron absorption, yet findings from a randomized controlled trial suggest that moderate calcium supplementation does not adversely impact iron status. In a 12-week study, healthy premenopausal women consuming 1000 mg of calcium carbonate daily with meals showed no significant differences in ferritin, serum iron, total iron-binding capacity (TIBC), transferrin saturation, hemoglobin, or hematocrit compared to controls. At baseline, plasma ferritin concentrations positively correlated with heme-iron intake, serum iron levels, transferrin saturation and hemoglobin concentration while negatively correlated with TIBC (r = −0.42, *p* < 0.001). These findings confirm that calcium supplementation does not interfere with heme iron absorption and iron homeostasis [34].

Hepcidin, a peptide hormone produced by the liver, tightly regulates iron absorption by inhibiting ferroportin activity. During iron sufficiency or overload, hepcidin production increases, blocking iron release into circulation, while reduced hepcidin levels during iron deficiency or hypoxia facilitate enhanced absorption through upregulation of DMT1 and FPN1 [28,35]. It is possible that communication between the gut and enterocyte (regarding iron concentrations) may also serve as a feedback inhibitory loop effect through hepcidin.

### 4.1. Implications of Heme Iron for Improving Iron Status

Understanding the differences in heme and non-heme iron absorption mechanisms underscores the advantages of heme iron for addressing iron deficiency. With minimal interference from dietary inhibitors and reduced side effects, heme iron presents a promising solution for supplementation strategies targeted at improving iron status across diverse populations. Supplementation with heme iron represents an opportunity to overcome many limitations for non-heme iron for a wide range of dietary needs, including supplements, fortified foods and medical foods.

Heme iron offers significant advantages in terms of gastrointestinal tolerability compared to non-heme iron supplements. Its high bioavailability allows it to be effective at lower doses, reducing the risk of gastrointestinal (GI) side effects commonly associated with high-dose non-heme iron supplements, such as ferrous sulfate or fumarate. Additionally, non-heme iron salts often result in unabsorbed iron remaining in the gut, leading to oxidative stress and symptoms like nausea, constipation, and abdominal pain [12]. In contrast, heme iron has a saturation threshold of approximately 15 mg, beyond which absorption does not increase significantly. This likely is an evolutionary adaptation to ensure minimal oxidative stress and reduced free iron accumulation in the gut and target tissues [11].

Research highlights that heme iron is less dependent on enhancers or environmental/dietary factors (i.e., vitamin C) for absorption, further contributing to its efficiency and tolerability. For example, a heme-based snack containing 7.2 mg of heme iron and 2.1 mg of non-heme iron per serving significantly improved iron status with optimal absorption. Further, combining heme iron with non-heme iron has been shown to improve the total absorption of dietary iron by up to 40% [18]. Opportunities exist to provide improved nutrition through the two separate but complementary nutrient uptake pathways—active and passive—which is a new strategy to reduce dose-related side effects [18].

### 4.2. Safety of Heme Iron

Heme iron offers improved bioavailability at a lower iron dosage, along with a reduced risk of adverse effects when consumed on a daily basis. Thus, heme iron, as an ingredient used for fortified foods and supplements, may represent a safer and potentially more effective option for long-term use and compliance [3,11].

Heme iron is a natural dietary component widely found in animal-based foods such as red meat, poultry, and seafood. It is considered Generally Recognized as Safe (GRAS) due to its ubiquitous presence in the food supply and its well-documented benefits in supporting adequate iron status. Based on sub-chronic toxicity studies, a No-Observed-Adverse-Effect-Level (NOAEL) for heme iron is 5% of dietary intake was stated in both male and female rats. This corresponds to an iron intake of approximately 58–75 mg/kg body weight per day for males, and 77–100 mg/kg for females. These levels are significantly higher than the NOAEL reported for elemental iron, which is estimated at 0.27 mg/kg body weight per day [36,37]. These findings underscore heme iron’s superior safety profile compared to non-heme iron salts, such as ferrous sulfate, which rapidly dissociate in the gut, contributing to gastrointestinal toxicity and systemic oxidative stress [12].

Heme iron supplementation has also been shown to cause fewer gastrointestinal side effects than traditional non-heme iron supplements. This advantage can be attributed to heme iron’s efficient absorption mechanism, which minimizes the presence of unabsorbed iron in the gastrointestinal tract. Studies report that heme iron supplementation is associated with adverse event rates comparable to placebo and significantly lower than those observed with ferrous sulfate or gluconate. For instance, Hoppe et al. [11] demonstrated that a low-dose heme iron intervention, using blood-based crispbread containing 7.2 mg heme iron per serving, effectively improved iron status while avoiding common side effects such as nausea, constipation, and abdominal discomfort.

Concerns remain regarding the broader dietary context where heme iron is consumed as part of red and processed meats. Epidemiological studies have linked red meat consumption to an increased risk of colorectal cancer and other chronic diseases. This association is partly attributed to heme iron’s ability to catalyze the formation of N-nitroso compounds (NOCs) and lipid peroxidation products, which are implicated in carcinogenesis [38,39]. Cooking methods such as grilling, frying, and smoking contribute to the formation of carcinogenic compounds like heterocyclic amines (HCAs) and polycyclic aromatic hydrocarbons (PAHs), further complicating the risk profile of red meat [40]. Importantly, these risks are specific to red meat, and not poultry, fish, and other seafood, suggesting that factors beyond heme iron—such as nitrates, nitrites, and cooking practices—play significant roles in carcinogenesis. Fish and poultry may be preferred sources of heme iron for dietary and supplement regimens [19,41].

The contribution of heme iron to oxidative stress, or the creation of nitroso- and carcinogenic compounds from cooking and grilling is likely to be minimal compared to other predominant components of animal foods and related lifestyle patterns which are also known to negatively impact health. Heme iron’s unique absorption mechanism supports a more predictable iron regulation in the body. For example, studies suggest that heme iron absorption is less sensitive to hepcidin activity than non-heme iron, enabling more efficient utilization of iron without exacerbating systemic oxidative stress. This characteristic makes heme iron a promising alternative for individuals who experience poor tolerability with traditional iron salts or chelates [4,6].

In conclusion, while heme iron is associated with significant safety and efficacy advantages, especially in controlled supplementation contexts, its broader dietary implications require careful consideration. Promoting emerging sources of heme iron from renewable, upcycled sources like seafood may help address global iron deficiency while mitigating the health risks linked to red meat consumption.

## 5. Improving Compliance with Iron Supplements

Supplemental iron is often prescribed to address deficiencies and nutritional insufficiencies [29]. However, supraphysiological doses commonly chosen for intervention are known to have negative side effects, including gastrointestinal complications such as constipation and nausea [12,29]. Such GI disturbances often lead to noncompliance and may also negatively impact oral nutritional intake [12,29]. An additional concern is the potential interference of high doses of iron with the absorption of other minerals, such as zinc, which may further enhance the production of free radicals [29].

And yet, supplementation remains an important contributor to overall iron intake for a large portion of the population. For example, a dietary intake study of 1385 female adolescents in Poland reported an average iron intake of 13 mg per day. Of this, as much as 60%—or 8 mg—came from non-heme iron sources such as fortified cereals, nuts, fruits, and vegetables. Less than 2 mg per day came from heme iron [42]. The mean intake of iron for all body weight types ranged from 12.6 to 13.7 mg per day, which is lower than the typical recommended intake of iron [42]. Other studies further demonstrate that inadequate iron intake compared to requirements remains a global, systemic issue.

### Lowering Iron Toxicity

Excessive iron supplementation or poor absorption of non-heme iron can lead to the accumulation of unabsorbed free iron in the gut, resulting in oxidative stress and significant intestinal damage [43]. The production of reactive oxygen species (ROS) is one of the primary mechanisms of this toxicity, as free iron reacts with hydrogen peroxide through Fenton chemistry to produce hydroxyl radicals. These ROS cause damage to intestinal epithelial cells, disrupt the gut barrier, and alter the composition of gut microbiota [12,44].

Unabsorbed iron may disrupt gut microbiota by reducing beneficial microbes and promoting pathogenic ones [12]. Beyond microbiome alterations, high levels of unabsorbed iron can directly damage the intestinal mucosa, contributing to conditions such as gastroduodenitis, intestinal barrier dysfunction, and inflammatory bowel disease [43].

The redox activity of free iron also poses systemic risks. Oxidative stress originating in the intestine can propagate systemically, contributing to cardiovascular disease, neurodegenerative conditions, and even colon cancer. Epidemiological studies have linked excessive iron intake with an elevated risk of colorectal cancer, mediated by lipid peroxidation products and *N*-nitroso compound formation catalyzed by free iron [38]. High doses of oral iron have also been associated with blackened stools and teeth, further highlighting the physical manifestations of toxicity.

Free iron may interfere with zinc absorption and impair gut function [29]. These effects underscore the necessity of carefully regulating iron supplementation to avoid creating toxic conditions in the gastrointestinal tract and beyond.

From a toxicological perspective, traditional non-heme iron salts, such as ferrous sulfate and ferrous gluconate, may be problematic because they rapidly dissociate in the gut, releasing a high concentration of free iron. Studies have found that up to 90% of non-heme iron from these supplements remains unabsorbed, resulting in oxidative stress and inflammation in intestinal epithelial cells [33]. Furthermore, overuse of high-dose iron supplements is thought to disrupt patient compliance due to the severity of gastrointestinal side effects, such as constipation, nausea, and abdominal pain, further limiting their effectiveness in addressing iron deficiency anemia [12].

In conclusion, while iron supplementation is critical for addressing iron deficiency anemia, excessive doses or poorly absorbed forms of iron can result in significant toxicity in the gut and beyond. A more targeted approach to supplementation—such as the use of heme iron—can mitigate these risks while improving the efficacy and safety of iron therapy. Heme iron’s reduced reliance on high doses and its absorption efficiency make it a promising alternative for addressing the complications associated with iron overload.

## 6. Innovations Beyond Non-Heme Iron Salts and Chelates

Non-heme iron salts and chelates were introduced in the early 20th century as food and supplement ingredients to address nutritional shortages during the war era, providing a mass-producible form of iron to meet regulatory, supply, and economic requirements [30]. Examples include ferrous gluconate, citrate, fumarate, and sulfate, which are synthesized through the purification of mined Earth minerals and processed using industrial techniques. Over the years, studies have documented both the benefits and limitations of these industrially produced iron salts and chelates.

Common ferrous and ferric iron salts include ferrous gluconate, citrate, and sulfate, with ferrous iron exhibiting higher bioavailability than ferric iron [8]. Among iron supplements, ferrous sulfate is the most widely used, providing 20% elemental iron [30,45]. Ferrous fumarate contains 33% elemental iron, while ferrous gluconate contains 12% elemental iron [30]. Both fumarate and gluconate release iron more slowly than ferrous sulfate [33]. Slow-release tablets, designed to reduce gastrointestinal side effects, improve tolerance through controlled release mechanisms achieved by polymeric matrices or tablet coatings [46]. However, their efficacy depends on gastric emptying, as incomplete dissolution of the tablet in the stomach reduces iron absorption [46].

Iron bisglycinate chelate provides improved bioavailability compared to ferrous salts by avoiding insoluble compound formation with dietary inhibitors like oxalates, phytates, and tannins [45]. This is due to its unique composition, wherein the ferrous cation is coupled with two glycine molecules [45]. Equal doses of iron bisglycinate are expected to yield more significant improvements in iron status compared to ferrous sulfate [45].

Non-heme iron absorption is influenced by numerous factors that impact real-life benefits. Some of these factors include the form of iron, the timing of administration, whether taken with food or an empty stomach, the composition and amount of food, whether taken with Vitamin C, etc. [1]. Additional individual variables—such as age, gender, menstrual cycle timing, health conditions, other nutrient deficiencies, nutrient interactions, and medications—further impact absorption [1]. These factors greatly complicate the accurate measurement of iron status, which remains the primary diagnostic tool for identifying iron deficiency or insufficiency [1].

The complex system of non-heme iron metabolism has been reviewed by others [43]. For non-heme iron, duodenal enterocytes absorb inorganic dietary iron via divalent metal transporter 1 (SLC11A2 or DMT1) after reduction by membrane-bound ferrireductases (DCYTB), the enzymes that reduce ferric iron to ferrous iron, often as a by-product of other pathways (Figure 1) [43]. The transport through DMT-1 is competitive with calcium, magnesium and other minerals in food. At maximum, daily absorbed iron, in the range of 1–3 mg, represents only a fraction of the total body iron. In all instances, iron must be replenished for all people daily, due to the recycling of heme from erythrocytes by reticuloendothelial macrophages, which provides the main fraction of circulating iron. Thus, while iron status is an important part of the iron nutrient picture, stored iron in the form of ferritin and hemoglobin provides a fuller picture compared to serum iron.

Iron salts and chelates pose significant limitations in addressing nutritional challenges due to their tendency to expose high concentrations of free iron in the gastrointestinal tract, which often remains unabsorbed. This results in ongoing concerns about the absorption, efficacy, and safety of these iron forms [45]. For instance, ferrous sulfate, one of the most commonly used iron supplements, provides 20% elemental iron but dissociates rapidly into free, unbound iron in the gut, which can exacerbate toxicity [30,45]. The absorption, distribution, metabolism, and excretion (ADME) characteristics of iron salts and chelates vary considerably. Some ferrous salts and chelates claim to offer a slower release compared to ferrous sulfate or ferric salts, theoretically reducing side effects associated with rapid dissociation. However, data on these claims remain mixed. “Slow-release” or gentler iron forms still demonstrate similar absorption rates, leading to adverse event rates between 20 and 30% [33]. Iron amino acid chelates, which hydrolyze faster and more extensively in the small intestine, present a greater magnitude of dissociation compared to heme iron [8].

**Figure 4:**
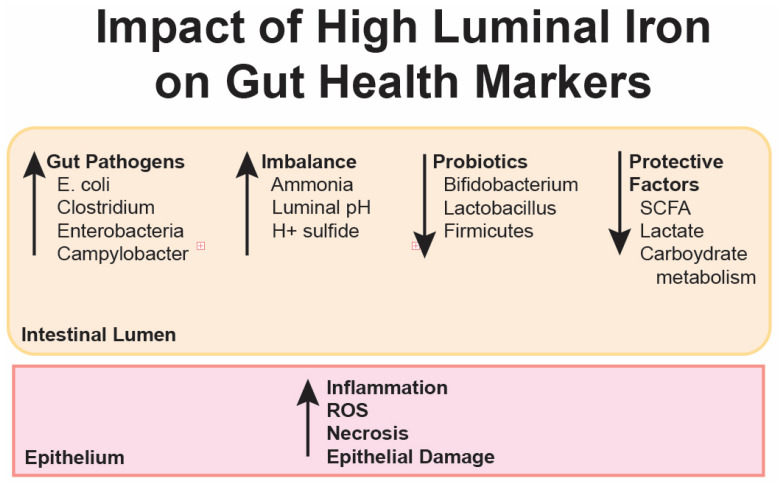
The complex relationship between gut microbiota and iron homeostasis, highlighting iron’s pivotal role in shaping microbial composition and activity in the gastrointestinal tract. Iron availability influences the balance between commensal and pathogenic microorganisms, with unabsorbed iron promoting the proliferation of pathogens such as *Salmonella* and *Escherichia coli*, while limiting beneficial bacteria like *Lactobacillus* and *Bifidobacterium*. Beneficial microbes contribute to intestinal health by maintaining barrier integrity and producing metabolites such as short-chain fatty acids (SCFAs), which enhance iron absorption. However, excessive free iron disrupts microbial balance, resulting in dysbiosis, oxidative stress, and inflammation in the gut. The figure emphasizes how iron supplementation strategies must carefully regulate iron availability to preserve gut microbial health and prevent adverse effects on the intestinal epithelium (adapted from [43]).

Excess oral iron can lead to triggered hepcidin response, inflammation, and production of reactive oxygen species (ROS) in intestinal epithelial cells (Figure 4). These can trigger ferroptosis, apoptosis, and necrosis [6]. As the primary barrier to high iron concentrations, epithelial cells suffer damage when exposed to excess free iron, resulting in inflammation and oxidative stress [28]. Mitochondrial damage and endoplasmic reticulum dysfunction are also hallmarks of excess cellular iron [44]. Simultaneously, free iron can trigger the growth of intestinal pathogens, resulting in gastrointestinal symptoms and increased levels of intestinal inflammation. The easier dissociation of iron in the gut, especially under conditions of high concentration, significantly increases the likelihood of severe intestinal inflammation [28].

Iron amino acid chelates are believed to interact less with dietary inhibitors like oxalates, phytates, and tannins, though direct comparative evidence is limited [45]. Studies indicate that iron chelated to purified amino acids such as glycine may enhance absorption slightly compared to conventional iron salts. However, comparative analyses consistently show that equivalent doses of iron bisglycinate and ferrous sulfate produce comparable improvements in iron status [45].

## 7. The Red Meat Paradox

Adequate iron can be consumed from food sources; however, the amount needed to meet the recommended intake for some individuals may be problematic. Heme iron is the most bioavailable form of dietary iron, primarily found in red meat, poultry, and fish [7]. While these are sources of heme iron, increased consumption is a concern for other health risks. Epidemiological studies have reported an association between high red and processed meat intake and an increased risk of colorectal cancer [38,39]. The International Agency for Research on Cancer (IARC) classified processed meats as Group 1 carcinogens and red meat as probable carcinogens [38]. In contrast, other heme iron sources like poultry, fish, and pork do not show the same level of risk, indicating that factors beyond heme iron, such as nitrates, nitrites, and cooking methods producing carcinogenic compounds like heterocyclic amines (HCAs), may play a role [47,48].

One proposed mechanism involves heme iron’s ability to catalyze the formation of N-nitroso compounds (NOCs) and lipid peroxidation products, both of which have been linked to carcinogenesis [39,49]. Additionally, the presence of nitrates, nitrites, and heterocyclic amines (HCAs) in processed and high-temperature cooked meats could further contribute to the cancer risk [50,51]. The cooking method may be another layer of complexity and further impact the risk of cancer from increased meat consumption. Heterocyclic amines (HCAs), particularly heterocyclic aromatic amines (HAAs) and polycystic aromatic hydrocarbons (PAHs), are potent mutagens formed during high-temperature cooking [50,51,52]. Grilling, pan frying and boiling meat have all been found to increase the rates of certain types of cancer [40].

Epidemiologic studies have linked increased consumption of red meat and processed meat with colon cancer. Bastide et al. found a positive association between colorectal cancer and heme iron intake via red meat, but this may reflect broader dietary patterns rather than heme iron alone [39]. While red and processed meats have been associated with an increased risk of colorectal cancer, evidence suggests that other sources of heme iron, such as fish, chicken, and pork, do not carry the same level of risk. A prospective cohort study by English et al. [41] found that while high consumption of red and processed meats was linked to an elevated risk of colorectal cancer, chicken consumption showed a weak negative association, and fish consumption had no significant impact [41].

A meta-analysis by Zhao et al. found an association between elevated ferritin levels and increased risk of Type 2 Diabetes (T2D), particularly in populations with iron-rich diets. However, the study had limitations, including inconsistent methodologies and confounding variables, making it difficult to establish causation [53]. These findings indicate that the increased cancer risk associated with red and processed meats may be influenced by factors beyond heme iron, such as nitrates, nitrites, and cooking methods that produce carcinogenic compounds like heterocyclic amines (HCAs) and polycyclic aromatic hydrocarbons (PAHs). In contrast, heme iron from fish, chicken, and pork appears to be safer and may provide a valuable dietary source of iron without the associated cancer risks.

## 8. Opportunities for Heme Iron in Nutrition

Supplemental iron is commonly prescribed to address deficiencies and nutritional insufficiencies [29]. In the United States, doses of 65 mg or more of elemental iron are frequently used for intervention. Approximately 14–18% of Americans take iron supplements, with the highest prevalence among pregnant (72%) and lactating women (60%) [12]. However, high-dose iron supplementation is often associated with gastrointestinal (GI) side effects. Due to limited absorption, up to 90% of supplemental iron remains in the small intestine, leading to side effects such as gastroduodenitis, nausea, vomiting, abdominal pain, dyspepsia, constipation, bloating, diarrhea, and dark stools [12,54]. Individuals with pre-existing gastrointestinal conditions or chronic inflammatory diseases may experience more pronounced side effects and reduced iron absorption [12].

Recent studies highlight the frequency and severity of GI side effects from oral iron supplementation. A meta-analysis of over 10,000 patients reported high rates of GI adverse effects across different iron formulations: ferrous fumarate (43%), ferrous gluconate (31%), and ferrous sulfate (30%) [54]. Another meta-analysis of 10,695 patients found side effect rates of 23% for ferrous glycine sulfate, 31% for ferrous gluconate, 32% for ferrous sulfate, and 47% for ferrous fumarate [12]. Conventional iron forms such as ferrous sulfate and ferrous fumarate were associated with a 30–45% frequency of adverse events, while chelated or glycinated iron forms showed a slightly lower yet significant incidence of 20–30% [12]. As expected, the severity of these effects tends to be dose-dependent: higher doses correlate with increased prevalence and severity of adverse events [12].

Non-heme iron supplementation at higher doses can induce hepcidin response, serving as a feedback inhibitor to iron absorption [55]. For example, in women with low ferritin, hepcidin response led to reduced iron absorption by 35–45% on the second day of administration of oral iron. Hepcidin response is also associated with iron overload. This has led to informal recommendations for alternate-day oral dosing or bolus IV in clinical iron deficiency, routines which may hinder compliance with treatment.

Heme iron, which is bound within porphyrin-heme in animal foods, is shown to offer improved tolerability. Research suggests that heme iron has a saturation threshold in the body of approximately 15 mg, beyond which absorption does not significantly increase [11]. For example, small doses of heme iron delivered via blood-based crisp bread (7.2 mg of heme iron and 2.1 mg of non-heme iron per serving) improved iron status within the optimal absorption range [11]. Furthermore, heme iron may enhance the overall absorption of non-heme iron when co-administered. For example, adding a small amount of heme iron to non-heme iron supplementation improved total iron absorption by approximately 40%. This suggests potential benefits for individuals with conditions such as short gut syndrome, where minimizing dose-related side effects is critical [29].

In conclusion, while oral iron supplements are widely used, their tolerability remains a significant concern, particularly at high doses. Heme iron appears to offer advantages in terms of absorption efficiency and reduced GI side effects. Future research should further explore optimal dosing strategies and the role of dietary heme iron in supporting iron status with minimal adverse effects.

## 9. Conclusions

Iron deficiency anemia (IDA) remains a pervasive global health challenge, affecting millions of individuals worldwide. Addressing this issue requires effective and sustainable solutions that balance efficacy, safety, and long-term feasibility. This review has highlighted the critical distinctions between heme and non-heme iron, emphasizing the superior bioavailability, tolerability, and sustainability of heme iron compared to its non-heme counterparts.

Heme iron’s efficient absorption mechanism, minimal interference from dietary inhibitors, and reduced gastrointestinal side effects make it an ideal candidate for addressing the limitations of traditional non-heme iron supplements. Incorporating heme iron into dietary strategies not only enhances overall iron uptake but also offers a safer alternative for populations vulnerable to the adverse effects of non-heme iron. Products like SalmoFer^®^, which utilize byproducts of the fish industry, underscore the potential of combining nutritional efficacy with environmental sustainability [11,19].

Despite these advantages, the broader dietary context in which heme iron is consumed, particularly through red and processed meats, presents challenges that require careful navigation. Red meat remains a potent source of bioavailable iron, yet its association with colorectal cancer and other chronic diseases necessitates the exploration of safer alternatives, from fish or poultry. Renewable produced alternatives provide an opportunity to mitigate health risks while meeting global iron demands [38,39].

Investigating the potential synergies between heme and non-heme iron in supplementation strategies could offer a balanced approach to improving iron bioavailability while reducing side effects. Additionally, exploring the impact of heme iron on gut microbiota, inflammation, and long-term health outcomes will strengthen its role in iron deficiency interventions [43]. Future research should prioritize refining heme iron supplements to enhance production consistency, optimize clinical efficacy, and expand accessibility to underserved populations.

In conclusion, balancing heme with non-heme iron in the diet is a science-based way to address iron insufficiency and deficiency in humans using diet and supplementation. Both heme and non-heme iron can play a pivotal role in addressing iron malnutrition globally.

## Figures and Tables

**Table 1:** Reference ranges for blood markers of iron status [1,3,12,20].

	U.S.		Global	
	Men	Women	Men	Women
**Ferritin** (ng/mL)	30–300	12–150	>15	>15
**Serum Iron** (µg/dL)	65–175	50–170	10–30	10–30
**Total Iron Binding Capacity** (µg/dL)	250–450		250–450	
**Transferrin Saturation** (%)	20–50%		20–50%	
**Hemoglobin** (g/dL)	14–17	12–15	>13	>12
**Hematocrit** (%)	38–49	35–45	38–48	35–45

## Data Availability

No new data were created or analyzed in this study.
